# Chromatin Regulation by HP1γ Contributes to Survival of 5-Azacytidine-Resistant Cells

**DOI:** 10.3389/fphar.2018.01166

**Published:** 2018-10-16

**Authors:** Satoshi Imanishi, Tomohiro Umezu, Chiaki Kobayashi, Tomohiko Ohta, Kazuma Ohyashiki, Junko H. Ohyashiki

**Affiliations:** ^1^Institute of Medical Science, Tokyo Medical University, Tokyo, Japan; ^2^Department of Translational Oncology, St. Marianna University Graduate School of Medicine, Kawasaki, Japan; ^3^Department of Hematology, Tokyo Medical University, Tokyo, Japan

**Keywords:** drug resistance, azacytidine, histone modification, heterochromatin protein 1, bromodomain inhibitor, leukemia

## Abstract

Recent investigations of the treatment for hematologic neoplasms have focused on targeting epigenetic regulators. The DNA methyltransferase inhibitor 5-azacytidine (AZA) has produced good results in the treatment of patients with myelodysplastic syndromes. The mechanism underlying its pharmacological activity involves many cellular processes including histone modifications, but chromatin regulation in AZA-resistant cells is still largely unknown. Therefore, we compared human leukemia cells with AZA resistance and their AZA-sensitive counterparts with regard to the response of histone modifications and their readers to AZA treatment to identify novel molecular target(s) in hematologic neoplasms with AZA resistance. We observed an a decrease of HP1γ, a methylated lysine 9 of histone H3-specific reader protein, in AZA-sensitive cells after treatment, whereas AZA treatment did not affect HP1 family proteins in AZA-resistant cells. The expression of shRNA targeting HP1γ reduced viability and induced apoptosis specifically in AZA-resistant cells, which accompanied with down-regulation of ATM/BRCA1 signaling, indicating that chromatin regulation by HP1γ plays a key role in the survival of AZA-resistant cells. In addition, the amount of HP1γ protein in AZA-sensitive and AZA-resistant cells was decreased after treatment with the bromodomain inhibitor I-BET151 at a dose that inhibited the growth of AZA-resistant cells more strongly than that of AZA-sensitive cells. Our findings demonstrate that treatment with AZA, which affects an epigenetic reader protein and targets HP1γ, or a bromodomain inhibitor is a novel strategy that can be used to treat patients with hematopoietic neoplasms with AZA resistance.

## Introduction

In recent years, the treatment for hematologic neoplasms has focused on targeting epigenetic regulators, such as DNA methyltransferases, histone deacetylases, and bromodomain proteins, as well as tyrosine kinases and proteasomes. The DNA methyltransferase inhibitor AZA has produced good results in the treatment of patients with myelodysplastic syndromes. Because of its DNA demethylation activity, most studies of the mechanism underlying the pharmacological activity of AZA have focused on the reactivation of tumor suppressor genes via demethylation of DNA in the promoter region (Lund et al., 2014; Tsujioka et al., 2015; Achille et al., 2016). However, recent studies have demonstrated the involvement of aberrant RNA metabolism (Aimiuwu et al., 2012), disruption of ribosome biogenesis (Moss, 2011), DNA damage response (Stresemann and Lyko, 2008), and histone modifications (Komashko and Farnham, 2010; Grövdal et al., 2014) in AZA activity, suggesting that the mechanism underlying its pharmacological activity is more complex than initially considered.

In parallel with research on the pharmacological activity of AZA, other studies have revealed the molecular mechanisms involved in AZA resistance. The importance of down-regulation of pyrimidine salvage in AZA resistance, which results in reduced phosphorylation of AZA, is well established (Saunthararajah, 2013; Imanishi et al., 2014; Valencia et al., 2014). The correlation of aberrantly high expression of ribosomal RNA with AZA resistance and the involvement of constitutive activation of the DNA damage response and DNA hypomethylation in AZA resistance have also been reported (Imanishi et al., 2014; Monika Belickova et al., 2016). However, chromatin regulation in AZA-resistant cells is still largely unknown.

In this study, we investigated the dynamics of histone modifications and their reader proteins in AZA-resistant cells to examine the possibility that they may be the novel targets in AZA-resistant hematologic neoplasms.

## Materials and Methods

### Cells and Reagents

U937 and HL-60 cells were purchased from ATCC (Manassas, VA, United States). AZA-resistant cell lines (R-U937 and R-HL-60) were originally created in our laboratory from U937 and HL-60 cells, respectively (Imanishi et al., 2014).

5-Azacytidine was purchased from Wako Pure Chemical Industries (Osaka, Japan) and I-BET151 from Selleck Chemicals (Houston, TX, United States). The antibodies specific for HP1α, HP1β, HP1γ, and β-actin were purchased from Abcam (Cambridge, United Kingdom). The antibodies specific for BRCA1 phosphorylated at Ser 1423 (p-BRCA1), total BRCA1 and total ATM were from Santa Cruz Biotechnology (Dallas, TX, United States) and anti ATM phosphorylated at Ser 1981 (p-ATM) antibody was from R&D Systems (Minneapolis, MN, United States). Anti PARP1 antibody was from Cell Signaling Technologies (Danvers, MA, United States). The secondary antibodies, namely horseradish peroxidase–labeled anti-mouse IgG antibody and horseradish peroxidase–labeled anti-rabbit IgG antibody, were purchased from GE Healthcare (Buckinghamshire, United Kingdom).

### Cell Culture and Chemical Reagent Treatment

U937, HL-60, R-U937, and R-HL-60 cells were incubated in RPMI1640 medium (Life Technologies Inc., Carlsbad, CA, United States) including 10% inactivated fetal bovine serum and 1% penicillin/streptomycin (Life Technologies Inc., Carlsbad, CA, United States). For treatment with reagents, cells were collected by centrifugation and suspended at 1 × 10^5^ cells/ml in fresh medium with the reagents or 0.01% DMSO as the vehicle. Cell viability was measured using the Cell Counting Kit-8 (Dojindo, Kumamoto, Japan) as previously described (Umezu et al., 2013).

### Western Blotting

Western blotting of HP1α, HP1β, HP1γ, p-BRCA1, total BRCA1, p-ATM, total ATM, PARP1 and β-actin was performed using whole cell lysate as previously described (Umezu et al., 2013). Briefly, the membranes were probed with antibodies directed against HP1α (1:500), HP1β (1:500), HP1γ (1:500), p-BRCA1 (1:500), total BRCA1 (1:500), p-ATM (1:200), total ATM (1:200), PARP1 (1:500), or β-actin (1:1000) and then treated with the appropriate secondary antibodies.

### Establishment of Doxycycline-Inducible shHP1γ Transfected Cells

The plasmid used in this study, CS-RfA-shHP1γ#1-ETBsd, was prepared as previously reported (Wu et al., 2015). This plasmid expresses shHP1γ by treatment with doxycycline. Lentivirus containing CS-RfA-shHP1γ#1-ETBsd or CS-RfA-ETBsd as a negative control was prepared using the Single Shot Lenti-X Packaging Kit (Takara Bio, Ohtsu, Japan) and Lenti-X 293 cells (Takara Bio). U937, HL-60, R-U937, and R-HL-60 cells were suspended in the medium containing lentivirus at 1 × 10^5^ cells/ml, and medium containing fresh lentivirus was added at 24 and 48 h. After incubation for 72 h, the cells were incubated in fresh medium containing 10 μg/ml blasticidin for 7 days. The surviving cells were maintained in medium containing 10 μg/ml blasticidin. To induce the expression of shHP1γ, the cells were treated with 1 μg/ml doxycycline.

### Quantitative RT-PCR

Quantitative RT-PCR was performed as previously described (Ohyashiki et al., 2005). Total RNA was purified from AZA treated cells, shHP1γ transfected cells or negative control cells incubated in the presence of 1 μg/ml doxycycline for 96 h. TaqMan gene expression assays (Life Technologies Inc., Carlsbad, CA, United States) were used for *CBX5* coding HP1α (Hs01127577_m1), *CBX1* coding HP1β (Hs01080635_g1), and *CBX3* coding HP1γ (Hs04234989_g1). TaqMan Pre-Developed Assay Reagent (Life Technologies Inc., Carlsbad, CA, United States) was used for *ACTB*. The expression level of *CBX1*,*3* and *5* relative to the *ACTB* expression level was determined by the ΔCT method.

### Flow Cytometric Analysis of Apoptosis

The FITC Annexin V Apoptosis Detection Kit I (BD Biosciences, San Jose, CA, United States) was used. Cell lines treated with doxycycline for 4 days were suspended in binding buffer and incubated with FITC-labeled annexin V and propidium iodide in the dark.

Flow cytometric measurements were performed on a BD Accuri C6 Flow Cytometer (BD Biosciences, San Jose, CA, United States). A 488-nm blue laser was used for excitation, and signals were detected using the FL1 channel (533 nm) for FITC and the FL2 channel (585 nm) for propidium iodide. The signals of 30,000 events were obtained. Analyses of the obtained data were performed by using C6 software version 1.0 (BD Biosciences, San Jose, CA, United States).

### Statistical Analyses

For statistical analyses, two-way ANOVA followed by the *t*-test were performed by using GraphPad PRISM 6 software (GraphPad Software Inc., La Jolla, CA, United States); *p* < 0.05 was considered significant. Data are shown as mean ± SD in the figures, and they represent the results obtained from three independent experiments.

## Results

### AZA Treatment Affected HP1 Family Proteins in AZA-Sensitive Cells but Not in AZA-Resistant Cells

**FIGURE 1:**
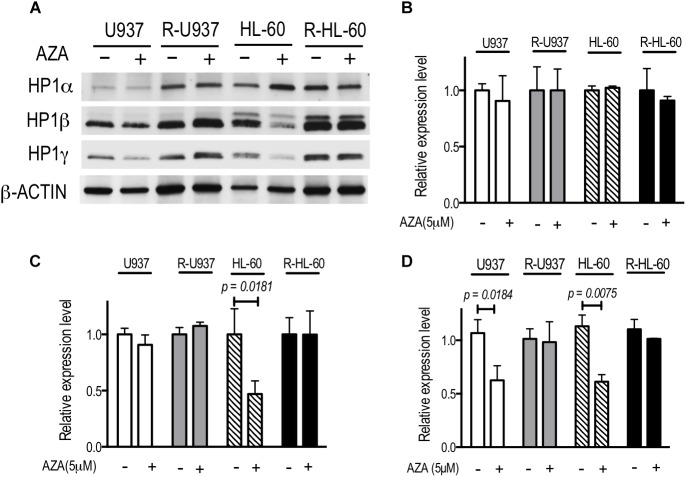
**(A)** The protein expression of HP1 family members after AZA treatment at 5 μM for 72 h in U937, R-U937, HL-60, and R-HL-60 cells. Typical blots from a representative experiment are shown. The experiments were repeated three times. **(B–D)** Relative mRNA expression of HP1α **(B)**, β **(C)**, and γ **(D)** after incubation with ( + ) or without (–) 5 μM AZA for 72 h in U937, R-U937, HL-60, and R-HL-60 cells. The expression level was normalized with *ACTB* mRNA expression. The means ± SD from three independent experiments are shown. *P*-values < 0.05 were indicated.

To investigate the chromatin regulation in AZA-resistant cells, we focused on HP1 proteins, the specific readers of di- or tri-methylated lysine 9 of histone H3 (H3K9), because previous studies showed that AZA treatment affected on the modifications of H3K9 (Grövdal et al., 2014; Tobiasson et al., 2017). The amounts of HP1α protein in U937, R-U937, HL-60, and R-HL-60 were not affected by AZA treatment (**Figure 1A**). In HL-60 cells treated with 5 μM AZA for 72 h, a four-fold decrease of HP1β was detected, whereas AZA treatment had no clear effect on the amount of HP1β in U937, R-U937, and R-HL-60 cells. Although a remarkable decrease in the amount of HP1γ was found in both U937 cells and HL-60 cells after AZA treatment, no such changes were detected in R-U937 and R-HL-60 cells. In HP1α mRNA expression, we did not detect any change in U937 cells, HL-60 cells, R-U937, and R-HL-60 cells after 5 μM AZA treatment for 72 h (**Figure 1B**). HP1β mRNA expression in U937 cells, R-U937 and R-HL-60 cells was not affected by 5 μM AZA treatment for 72 h, while that in HL-60 cells was significantly reduced after 5 μM AZA treatment for 72 h (**Figure 1C**). The mRNA expression of HP1γ was decreased in U937 cells and HL-60 cells, but not in R-U937 cells and R-HL-60 cells, after treatment with 5 μM AZA for 72 h indicating that AZA treatment repressed the transcription of HP1γ mRNA (**Figure 1D**). These results indicated that AZA treatment disrupted chromatin regulation via the methylated H3K9/HP1γ axis in AZA-sensitive cells but not in AZA-resistant cells.

### Targeting HP1γ Induced Apoptosis Specifically in AZA-Resistant Cells

**FIGURE 2:**
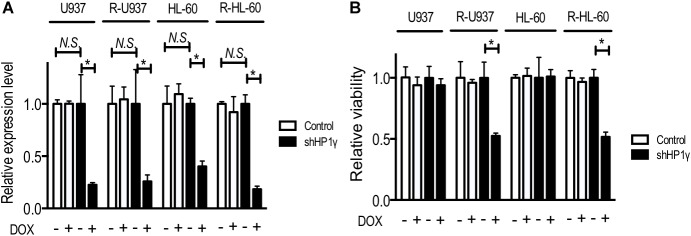
**(A)** Relative mRNA expression of HP1γ after incubation with (+) or without (–) 1 μg/ml doxycycline (DOX) for 72 h in control plasmid–infected cells (white bars) and shHP1γ-infected cells (black bars). The means ± SD from three independent experiments are shown. ^∗^*p* < 0.0001. **(B)** The relative viability of control plasmid–infected cells (white bars) and shHP1γ-infected cells (black bars) after incubation with (+) or without (–) 1 μg/ml DOX for 96 h. The means ± SD from three independent experiments are shown. ^∗^*p* < 0.0001.

**FIGURE 3:**
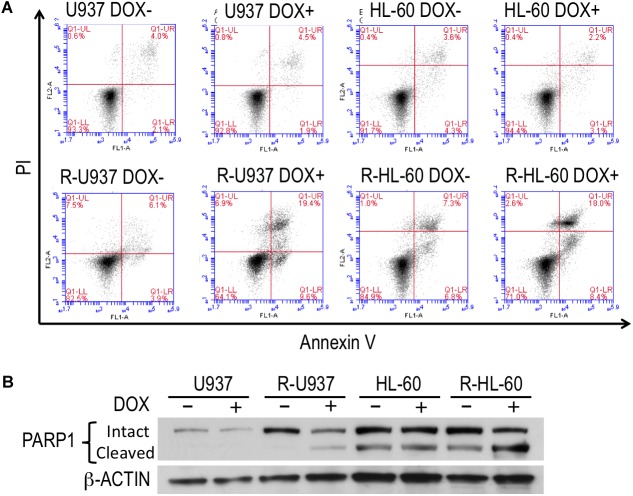
**(A)** FACS analysis of annexin V/propidium iodide double staining in shHP1γ-infected cells after incubation with (DOX+) or without (DOX–) 1 μg/ml doxycycline for 96 h. Typical plots from a representative experiment were shown. The experiments were repeated three times. **(B)** The PARP1 cleavage profiles in shHP1γ-infected cells after incubation with (DOX+) or without (DOX–) 1 μg/ml doxycycline for 96 h.

To clarify the role of HP1γ in AZA-resistant cells, we established cells expressing shRNA targeting HP1γ mRNA in a Tet-On system–dependent manner. As shown in **Figure 2A**, HP1γ mRNA significantly decreased after incubation for 72 h in the presence of 1 μg/ml doxycycline, demonstrating that HP1γ expression was successfully knocked down. The incubation with doxycycline for 96 h did not affect the viability of shHP1γ-infected U937 cells and shHP1γ-infected HL-60 cells or in negative control cells, whereas the growth of shHP1γ-infected R-U937 cells and shHP1γ-infected R-HL-60 cells was inhibited by doxycycline treatment (**Figure 2B**). In the analyses of annexin V/propidium iodide (PI) double staining, treatment with doxycycline for 96 h increased the annexin V–positive/PI–negative and annexin V–positive/PI–positive populations in shHP1γ-infected R-U937 cells and shHP1γ-infected R-HL-60 cells (**Figure 3A**, lower panels) but not in shHP1γ-infected U937 cells and shHP1γ-infected HL-60 cells (**Figure 3A**, upper panels). The increase of cleavage of PARP1 was detected in shHP1γ-infected R-U937 cells and shHP1γ-infected R-HL-60 cells treated with doxycycline, while the presence of doxycycline did not affect on the PARP1 cleavage in shHP1γ-infected U937 cells and shHP1γ-infected HL-60 cells (**Figure 3B**). These results suggested that targeting HP1γ was cytotoxic specifically in AZA-resistant cells.

### Targeting HP1γ Interfered ATM/BRCA1 Signaling in AZA-Resistant Cells

**FIGURE 4:**
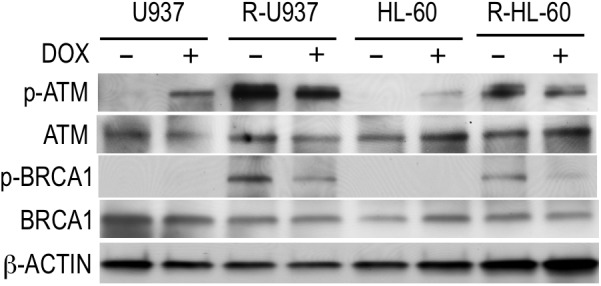
The profile of phosphorylation of ATM and BRCA1 in shHP1γ-infected cells after incubation with (+) or without (–) 1 μg/ml doxycycline for 72 h were shown. The typical result from three-times repeated experiments was shown.

To understand the role of HP1γ in AZA-resistant cells, we investigated the effect of HP1γ targeting on phosphorylation of ATM and BRCA1 proteins because these proteins are constitutively activated in AZA-resistant cells as we previously reported (Imanishi et al., 2014). As shown in **Figure 4**, p-ATM, but not p-BRCA1, was detected in shHP1γ-infected U937 cells and shHP1γ-infected HL-60 cells after incubation with doxycycline for 72 h indicating HP1γ was need to phosphorylate BRCA1 by active ATM in AZA-sensitive cells. P-ATM and p-BRCA1 were detected in shHP1γ-infected R-U937 cells and shHP1γ-infected R-HL-60 cells without doxycycline treatment. After treatment with doxycycline for 72 h, both of p-ATM and p-BRCA1 in shHP1γ-infected R-U937 cells and shHP1γ-infected R-HL-60 cells was decreased (**Figure 4**). These results suggested that HP1γ contributed to maintain the constitutive activation of ATM/BRCA1 signaling in AZA-resistant cells.

### Treatment With Bromodomain Inhibitor Decreased HP1γ Protein

Although our results indicated that HP1γ could be a novel molecular target in the treatment of myeloid neoplasms with AZA resistance, the small molecule targeting HP1γ is unfortunately unavailable. A relationship between sensitivity to bromodomain inhibitor and HP1γ expression was recently indicated (Knoechel et al., 2014), however, so we instead examined the effect of the bromodomain inhibitor I-BET151 on cell growth and HP1γ protein expression.

**FIGURE 5:**
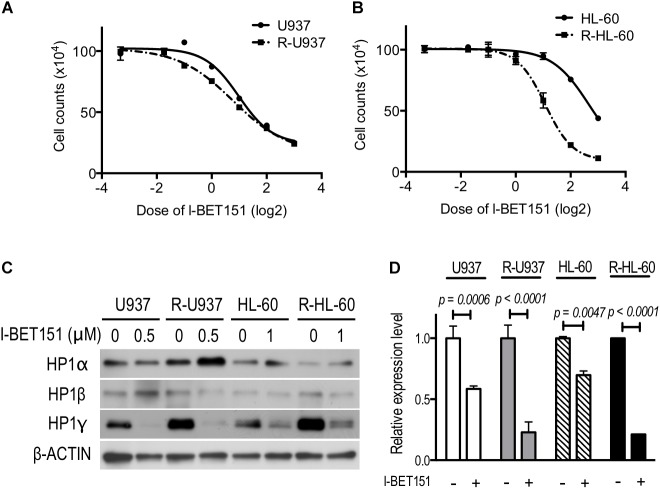
The drug response curve using the cell counts of **(A)** U937 (solid line) and R-U937 cells (dotted line) and **(B)** HL-60 (solid line) and R-HL-60 cells (dotted line) after incubation with 0.1, 0.3, 0.5,1, 2, 4, and 8 μM I-BET151 for 72 h. For each cell set, the mean ± SD from three independent experiments are shown. **(C)** Protein expression levels of HP1α, β, and γ in U937, R-U937, HL-60, and R-HL-60 cells after incubation with I-BET151 at indicated dose for 48 h. Typical blots from a representative experiment are shown. The experiments were repeated three times. **(D)** Relative mRNA expression of HP1γ after incubation with (+) or without (–) I-BET151 for 48 h. The concentration of I-BET151 was 0.5 μM for U937 and R-U937 cells and 1 μM for HL-60 and R-HL-60 cells. The mean ± SD from three independent experiments are shown. *P*-values < 0.05 were indicated.

We treated the cells with 0.1, 0.3, 0.5, 1, 2, 4, and 8 μM I-BET151 for 72 h. R-U937 cells (**Figure 5A**) and R-HL-60 cells (**Figure 5B**) showed higher sensitivity to I-BET151 in comparison with U937 cells and HL-60 cells, respectively. The IC50 values were 9.7 μM in U937, 6.0 μM in R-U937, 6.4 μM in HL-60 and 2.1 μM in R-HL-60. Because these results indicated that AZA-resistant cells were more sensitive to I-BET151 than their AZA-sensitive counterparts, we examined the effects of I-BET151 treatment on HP1 proteins (**Figure 5C**). The amount of HP1α protein was not affected by treatment with I-BET151 in U937, R-U937, HL-60, and R-HL-60 cells. A reduction of HP1β protein was found only in HL-60 cells treated with 1 μM I-BET151 for 48 h, whereas other cell lines showed no such changes. Treatment with I-BET151 at 0.5 μM for 48 h decreased HP1γ protein in U937 and R-U937 cells, and HP1γ protein in HL-60 and R-HL-60 cells was down-regulated after incubation with 1 μM I-BET151 for 48 h. In U937 and R-U937 cells, the decrease of HP1γ mRNA expression was detected after treatment with I-BET151 at 0.5 μM for 48 h as well as in HL-60 and R-HL-60 cells after treatment with I-BET151 at 1 μM for 48 h (**Figure 5D**). The repression of mRNA expression was more notable in R-U937 and R-HL-60 cells in comparison with their AZA-sensitive counterparts, indicating an important role of bromodomain proteins in HP1γ expression in AZA-resistant cells.

## Discussion

In the epigenetic regulation of gene expression and chromatin, epigenetic writers, erasers, and readers play roles through direct and indirect interactions (Fuks et al., 2000, 2003; Vire et al., 2006; Meier and Brehm, 2014). Therefore, drugs targeting an epigenetic regulator can also affect other regulator proteins or epigenetic marks. Grövdal et al. (2014) showed that AZA treatment reduced H3K27ac and H3K9ac and Tobiasson et al. (2017) reported increase of genes marked by H3K9me3 after treatment with AZA in addition to genome-wide DNA demethylation in CD34+ progenitor cells obtained from patients with myelodysplastic syndromes. However, the effects of AZA on epigenetic reader were left unknown. In this study, we found that AZA treatment caused the decrease of HP1γ in AZA-sensitive cells but not in AZA-resistant cells. These observations are the first evidence that AZA can affect an epigenetic reader and indicate that the pharmacological activity of AZA might involve disruption of heterochromatin regulation via the methylated H3K9/HP1γ axis.

Recently, Tobiasson et al. (2017) showed that treatment with 1 μM AZA for 24 h increased the expression of genes marked by H3K9me3 in CD34+ progenitor cells obtained from patients with myelodysplastic syndromes or myelodysplastic syndrome–related disease, but the changes in the H3K9me3 profile could explain only a minor part of changes in the gene expression profile. Interestingly, they also showed that changes in the gene expression profile did not correlate with DNA demethylation. Such dissociation between gene expression and epigenetic marks could be explained by the dysregulation of epigenetic readers such as HP1γ.

Members of the HP1 family, HP1α, β, and γ, recognize and bind to di- or tri-methylated H3K9, the epigenetic marks of heterochromatin, and function to compact heterochromatin (Zeng et al., 2010). However, some studies have shown that HP1γ, unlike HP1α and β, distributes to not only heterochromatin but also euchromatin (Lomberk et al., 2006; Zeng et al., 2010; Kwon and Workman, 2011). Lomberk et al. (2006) reported that phosphorylated HP1γ distributed on euchromatin interacts with Ku70, a regulatory protein in multiple nuclear processes. Another study by Vakoc et al. (2005) showed that HP1γ and H3K9me3 were associated with transcription elongation. Therefore, the down-regulation of HP1γ by AZA treatment indicates that some part of AZA activity might be caused by the disruption of multiple processes on euchromatin.

The knock-down experiment of HP1γ demonstrated that HP1γ-dependent chromatin regulation, which could not be replaced by HP1α and β, played an essential role in the survival of AZA-resistant cells. In this study, we showed that targeting HP1γ caused decrease of p-ATM and p-BRCA1 in AZA-resistant cells, suggesting that constitutive activation of ATM/BRCA1 signaling in AZA-resistant cells needs HP1γ function. Therefore, a possible explanation is that a decrease of HP1γ caused growth inhibition and apoptosis via down-regulation of the constitutively activated DNA damage repair response in AZA-resistant cells. On the other hand, because HP1γ activity is involved in multiple processes on euchromatin, another possibility is that the disruption of some euchromatin process caused growth inhibition and apoptosis. In AZA-sensitive cells, the phosphorylation of BRCA1, but not that of ATM, was inhibited by HP1γ targeting. It is reported that the recruitment of ATM to the DNA damage site requires HP1 family members (Goodarzi et al., 2008). Wu et al. (2015) demonstrated that BRCA1 retention at the DNA damage site depends on interaction of BARD1 and HP1γ. Our results in AZA-sensitive cells, which indicated the need of HP1γ to activate BRCA1 by ATM, well agree with these preceding studies. HP1γ targeting in AZA-sensitive cells might caused DNA damage and inhibited BRCA1 activation, while other DNA repair signaling such as PARP signaling might prevent growth inhibition and apoptosis in AZA-sensitive cells.

In spite of recent pharmaceutical developments focusing on the targeting of epigenetic readers (Dawson et al., 2012; Greschik et al., 2017; Zaware and Zhou, 2017), the small molecule targeting HP1 proteins is still unavailable. We found that I-BET151 down-regulated HP1γ at transcriptional level in both AZA-resistant and AZA-sensitive cells, indicating that I-BET151 can work as an inhibitor of HP1γ. Because the decrease of HP1γ showed a growth inhibitory effect specifically in AZA-resistant cells, the higher sensitivity to I-BET151 in AZA-resistant cells might involve the down-regulation of HP1γ. A recent study reported that T cell acute lymphoblastic leukemia cells with resistance against γ-secretase inhibitor showed higher expression of HP1γ and higher sensitivity to another bromodomain inhibitor, JQ1, than those without γ-secretase inhibitor resistance (Knoechel et al., 2014). These observations appear to indicate that the sensitivity to bromodomain inhibitors is related to expression of HP1γ or dependence on HP1γ. Further research into the relationship between bromodomain family euchromatin regulators and HP1γ is thus warranted.

Our approach had several limitations. Although the present results demonstrated that HP1γ-specific function(s) contributed to the survival of AZA-resistant cells, it is still unclear which function(s) played the key role in their survival. The relationship between dependence on HP1γ and sensitivity to BET inhibition should be confirmed in leukemia cells obtained from patients with AZA resistance.

Although the involvement of many cellular processes in AZA activity have been studied, its effects on epigenetic reader proteins have never been examined. In this study, we demonstrated that AZA affects epigenetic reader proteins, such as HP1γ. Our findings should help to improve our understanding of the mechanism(s) underlying AZA activity. One key finding is the importance of chromatin regulation by HP1γ in AZA-resistant cells. The agent targeting HP1γ still needs to be identified, but this study revealed the possibility that I-BET151 can work as a HP1γ inhibitor via down-regulation of HP1γ. HP1γ targeting or bromodomain inhibition could be a novel strategy for the treatment of hematologic malignancies with AZA resistance.

## Author Contributions

SI designated study, collected and analyzed data, and wrote the manuscript. TU and CK contributed data collections. TO provided the plasmids and advised for the experiments. KO and JO supervised the study. All authors reviewed the manuscript.

## Conflict of Interest Statement

KO received research support from Celegene KK, served as a consultant and advisor of Celegene KK, and received honoraria for lecture fees from Nippon Shinyaku.

The remaining authors declare that the research was conducted in the absence of any commercial or financial relationships that could be construed as a potential conflict of interest.

## Abbreviations

AZA: 5-azacytidine
HP1: heterochromatin protein 1
shHP1γ: shRNA targeting HP1γ
